# Is Anyone Home? A Way to Find Out If AI Has Become Self-Aware

**DOI:** 10.3389/frobt.2018.00017

**Published:** 2018-02-28

**Authors:** John Mark Bishop

**Affiliations:** ^1^TCIDA, Goldsmiths, University of London, London, United Kingdom

**Keywords:** machine consciousness, Turing test, ACT, beaviorism, artificial intelligence

Recent articles by Schneider and Turner (Turner and Schneider, 2017; Schneider and Turner, 2017) outline an artificial consciousness test (ACT); a new, purely behavioral process to probe subjective experience (“phenomenal consciousness”: tickles, pains, visual experiences, and so on) in machines; work that has already resulted in a provisional patent application from Princeton University (Turner and Schneider, in press). In light of the author’s generic skepticism of “consciousness qua computation” (Bishop, 2002, 2009) and Tononi and Koch’s “Integrated Information Theory”-driven skepticism regarding the possibility of consciousness arising in any classical digital computer (due to low ϕ^max^) (Tononi and Koch, 2015), consideration is given to the claimed sufficiency of ACT to determine the phenomenal status of a computational artificial intelligence (AI) system.

In science and science fiction, the hope is periodically reignited that a computer system will one day be conscious in virtue of its execution of an appropriate program; indeed, as far back as 2004, the UK funding body EPSRC awarded an “Adventure Fund” grant [GR/S47946/01] of around *£*500,000, to a team of “Roboteers and Psychologists” at the Universities of Essex and Bristol, with a goal of instantiating “machine consciousness” in a humanoid-like robot called Cronos. In addition, extant claims of “machine consciousness” have long been claimed in the scientific literature. (For example, in 2002, Kevin Warwick announced his “Cybernetic learning robots” to be “as conscious as a slug” (Warwick, 2002).)

Other proposals for conscious machines have ranged from the mere “functional consciousness” of Stan Franklin’s “Intelligent Distribution Agent” (Franklin, 2003) to the claim of “true conscious cognition” of [Pentti] “Haikonen’s Cognitivist Architecture” (HCA), an architecture that seeks to reproduce the processes of perception, inner imagery, inner speech, pain, pleasure, emotions, and the cognitive functions behind these. Haikonen has asserted that, when implemented with sufficient complexity, HCA will develop consciousness (Haikonen, 2012).

It is in this febrile atmosphere that Schneider and Turner (2017) highlight the importance of a test to ascertain machine consciousness as (i) it may be deemed morally improper to oblige such machines to “serve” humans; (ii) it could raise safety concerns; and (iii) it could impact on the viability of brain-implant technologies (Hampson et al., 2013). Hence, given the impact of an ACT result that ascribes consciousness to machine, it is critical that the test is both robust and accurate; in this context, Schneider and Turner explicitly clarify that passing ACT “… is *sufficient* but not *necessary* evidence for AI consciousness.”

Given that one of the most forceful indications that humans experience consciousness is that every adult can readily and quickly grasp concepts based on this quality, Schneider and Turner describe their ACT as follows:

[T]he ACT would challenge an AI with a series of increasingly demanding natural language interactions to see how quickly and readily it can grasp and use concepts and scenarios based on the internal experiences we associate with consciousness. At the most elementary level we might simply ask the machine if it conceives of itself as anything other than its physical self. At a more advanced level, we might see how it deals with ideas and scenarios such as those mentioned in the previous paragraph. At an advanced level, its ability to reason about and discuss philosophical questions such as ‘the hard problem of consciousness’ would be evaluated. At the most demanding level, we might see if the machine invents and uses such a consciousness-based concept on its own, without relying on human ideas and inputs.

Turner and Schneider claim that the above procedure is *sufficient* to establish consciousness in any “boxed-in” AI system (i.e., any AI not connected to the Internet); any AI that passes ACT will be conscious. But could a non-conscious AI machine cheat? Schneider and Turner (2017) specifically consider this question, outlining the following possible scenario:

Even today’s robots can be programmed to make convincing utterances about consciousness, and a truly superintelligent machine could perhaps even use information about neurophysiology to infer the presence of consciousness in humans. If sophisticated but non-conscious AIs aim to mislead us into believing that they are conscious for some reason, their knowledge of human consciousness could help them do so.

The solution here, so the author’s suggest, is simply to “box-in” the AI, denying it access to the Internet and “*… making it unable to get information about the world or act outside of a circumscribed domain*.”

But this methodology yields its own problems. For even if we cut off access to the Internet—and the AIs knowledge domain is restricted to “*prohibit it from gaining any knowledge of the world, especially information about conscious experience and neuroscience*”—we are led to the problem of explicitly identifying, *a priori*, precisely what knowledge needs to be circumscribed in this manner; alternatively, as one of the reviewers of this short piece pithily observed, if we cut off access to the Internet but allow access to the entire knowledge of the World Wide Web to be “pre-loaded” into the “box,” then the boxing-in idea would not appear to have added anything to the argument.

In addition, because the principle of computational multiple realizability states that, despite potential underlying physical differences in operation, it is possible to run the same functional program (e.g., Microsoft Word) on very different architectures (cf. Windows, MAC, SCO Unix, etc.), it is clear that were an AI’s successful responses merely generated by a suitably large “look-up table” (Block, 1981), it would *still* qualify as “passing” ACT.

Moreover, Schneider clarified at PTAI conference (Leeds, 2017) that ACT is robust to repeated use of exactly the same question set: if machine **M**, given a set **A** of *k* questions, responds with a set A* of *k* answers, in such a way that it is deemed to have passed ACT (and consciousness is ascribed to **M**), then if, posing exactly the same question set **A** to a second machine M*, generates exactly the same responses A*, then M* must also be deemed to have passed ACT; so construed, we note that the test is explicitly behaviorist in its conception.

Unfortunately, an unintended consequence of such behaviorism is that *any trivial machine* M**, hard coded to explicitly respond to question set **A** with responses A* (i.e., any machine simply programmed to output these *k* responses to those *k* questions), must also be deemed to pass ACT.

For these reasons, unless we are content to ascribe conscious sensation to a mere look-up table [of a list of acceptable questions and answers], it is not clear that ACT (or any purely behavioral test) can succeed as a *sufficient* test to establish phenomenal consciousness in an artificial system; furthermore, it is observed that objections to behaviorism along these lines date back at least to Chomsky’s sharp critique (Chomsky, 1959) of the cognitive vapidity of Skinner’s (Skinner, 1957) approach to language.

## Author Contributions

The author confirms being the sole contributor of this work and approved it for publication.

## Conflict of Interest Statement

The author declares that the research was conducted in the absence of any commercial or financial relationships that could be construed as a potential conflict of interest.
